# Shifting forward: Urban ecology in perspective

**DOI:** 10.1007/s13280-024-02007-6

**Published:** 2024-04-20

**Authors:** Steward T. A. Pickett, Niki Frantzeskaki, Erik Andersson, Aliyu Salisu Barau, Daniel L. Childers, Fushcia-Ann Hoover, Ariel E. Lugo, Timon McPhearson, Harini Nagendra, Selina Schepers, Ayyoob Sharifi

**Affiliations:** 1 Cary Institute of Ecosystem Studies, Box AB, Millbrook, NY 12545 USA; 2 Department of Human Geography and Spatial Planning, Faculty of Geosciences, Utrecht University, Vening Meinesz Building A, Princetonlaan 8a, 3485 CB Utrecht, The Netherlands; 3 Ecosystems and Environment Research Programme and Helsinki Institute of Sustainability Science (HELSUS), University of Helsinki, Viikinkaari 1, P.O. Box 65, 00014 Helsinki, Finland; 4 Stockholm Resilience Centre, Stockholm University, Stockholm, Sweden; 5 Research Unit for Environmental Sciences and Management, North-West University, Potchefstroom, South Africa; 6 Department of Urban and Regional Planning, Bayero University Kano, PMB 3011, Kano, Nigeria; 7 School of Sustainability, Arizona State University, POB 877904, Tempe, AZ 85287-7904 USA; 8 Department of Geography and Earth Sciences, University of North Carolina Charlotte, 9201 University City Blvd, Charlotte, NC 28223 USA; 9 International Institute of Tropical Forestry, USDA Forest Service, 1201 Calle Ceiba, Jardín Botánico Sur, Río Piedras, PR 00926-1115 USA; 10 Urban Systems Lab, The New School, 79 Fifth Ave, 16 Floor, New York, NY 10003 USA; 11 Stockholm Resilience Centre, Stockholm University, Stockholm, Sweden; 12 Beijer Institute of Ecological Economics, Royal Swedish Academy of Sciences, Stockholm, Sweden; 13 Centre for Climate Change and Sustainability, Azim Premji University, Burugunte Village, Bikkanahalli Main Road, Sarjapura, Bangalore, IN 562125 India; 14 Department of Environment and Sustainable Development, Stadsplein 1, 3600 Genk City, Belgium; 15 The IDEC Institute, Hiroshima University, 1-5-1 Kagamiyama, Higashi-Hiroshima, 739-8529 Japan; 16 School of Architecture and Design, Lebanese American University, Beirut, Lebanon

**Keywords:** Sustainability, Synthesis, Transdisciplinary, Urban ecology, Urbanization

## Abstract

The world has become urban; cities increasingly shape our worldviews, relation to other species, and the large-scale, long-term decisions we make. Cities *are* nature, but they need to align better with other ecosystems to avoid accelerating climate change and loss of biodiversity. We need a science to guide urban development across the diverse realities of global cities. This need can be met, in part, by shifts in urban ecology and its linkages to related sciences. This perspective is a “synthesis of syntheses”, consolidating ideas from the other articles in the Special Section. It re-examines the role of urban ecology, and explores its integration with other disciplines that study cities. We conclude by summarizing the next steps in the ongoing shift in urban ecology, which is fast becoming an integral part of urban studies.

## Introduction

Where is a whole field headed? In this “synthesis of syntheses” (the contributions to the Special Section “Shifts in Urban Ecology”), we step back and reflect on the process behind the Special Section. “Synthesizing” as an academic practice includes a remarkably diverse set of activities (Pickett et al. 2007), among them (1) the combination of disparate insights and approaches representing distinct disciplines or scales (Carpenter et al. 2009; Palmer et al. 2016); (2) bringing complementary data together in new ways (Gurevitch et al. 2018); (3) identifying neglected perspectives such as Indigenous ecological knowledge (Gewin 2021); and (4) conducting horizon or edge-work at the boundaries between fields (Hastrup 2014; Petryna 2022). The four papers in this Special Section address synthesis in complementary ways. First, Andersson et al. (2024) call for employing a wide variety of empirical approaches and sources of knowledge to improve the understanding and sustainability of urban systems. Frantzeskaki et al. (2024) detail how synthesis and integration can promote conceptual and theoretical advances or identify and contribute actionable knowledge to solving societal problems. Grove et al. (2024) show how synthesizing historical, policy, social, and environmental knowledge can help understand the persistence of racial and other social injustices in urban places and help social movements overcome them. Pickett et al. (2024) point to some of the past syntheses that have advanced urban ecological science and practice and the opportunities to extend the horizon of such syntheses.

The four papers in the Special Section describe shifts in the breadth of kinds of evidence the field needs to embrace (Andersson et al. 2024); the transformation of urban ecology from a curiosity-driven to an action-oriented field (Frantzeskaki et al. 2024); a shift from urban structure and form to various kinds of relationships with which the science must engage (Pickett et al. 2024); and finally, an evolution toward addressing injustices and inequities in urban places around the world (Grove et al. 2024). Each of the four papers represents a synthesis within its scope of interest. Jointly, these perspectives show how the general process of synthesis continues to refine urban ecology's capacity to contribute to positive urban futures. This paper, in turn, highlights key themes from the four papers in this Special Section. Furthermore, it demonstrates how the major themes addressed in these four syntheses relate to and reinforce one another. Given that urban ecology is evolving, our hope is that this synthesis of the key trends and distinct shifts in the structure, motivation, and justification of the field can enrich, inform, and provide deeper insights into human-nature interactions—an ecology “with the city” based on a new foundation that is more interactive and engaged (Pickett et al. 2021; Byrne 2022). The emerging, pluralistic approach may also help move urban ecology closer to the realm of action.

The proposed shifts are theoretical, conceptual, epistemological (how knowledge production is organized and realized), and axiological (identifying what “good” urban ecology science is and what knowledge is usable or applicable for). The shifts are, at least in part, an outcome of a field, and its scholars, now much more engaged with the concerns of communities, policymakers, and those responsible for the well-being of residents and ecosystems in the full variety of urban forms around the world. Rigorous urban social-ecological-technological systems (SETS) science (McPhearson et al. 2016, 2021a) must thus be complemented and informed by other forms of knowing (Andersson et al. 2024). In addition, urban SETS science requires translation into action situations in which social movements can play an important role (Frantzeskaki et al. 2021). This evolution is well underway, having moved from an “ecology in the city,” which studies the biophysical patches within a city, through “ecology of the city” with its focus on the entire city as a social-ecological system, to “ecology for the city,” which also applies social-ecological knowledge to solve problems identified by residents and decision makers (Childers et al. 2014; McPhearson et al. 2016; Zhou et al. 2021; Frantzeskaki et al. 2024). As the recently published IPCC report indicates (Dodman et al. 2022), cities contribute to carbon emissions but are also the places where many solutions to climate change and biodiversity loss have been conceived, tested, and proven effective. We need to employ a pluralistic and transformative mode for science to deal with the compound crises of the Anthropocene, including climate change, biodiversity loss, and deepening injustices, especially in the post-pandemic era. As the IPBES chair stated in 2019: “science has a transformative role in society”. We extend this implicit call to the transformative role of urban ecology in the future of cities.

Urban ecology is shifting to include the extended scale and diverse mechanisms of urbanization and its impact (Frantzeskaki et al. 2024; Pickett et al. 2024), a shift that can be traced back to the pioneering work by for example, Numata (1977), Goode (1989), Sukopp (2008) and Breuste (2009). The papers in this Special Section build on this foundation to show how urban ecology increasingly emphasizes relationships within and among urban places and to synthesize frameworks that support interdisciplinary work on the rapidly changing global urban realm. An example is the continuum of urbanity summarized in the Special Section (Pickett et al. 2024) and the shift towards including a deeper understanding of urban ecology through people-nature relations. As urban ecology pluralizes, the contexts in which its understanding is produced need to be better understood, and evidence needs to be collected, analyzed, and contrasted differently (Andersson et al. 2024). Against this background, urban ecology demonstrates an intention to transform the action-knowledge interface and feedback between science and policy and planning, thus becoming a transformative scientific inquiry connecting researchers/academics, communities, and planners (Frantzeskaki et al. 2024). In this way, urban ecology will need to shift in a way that more explicitly considers issues of equity and justice as critical to how science is produced. This includes going beyond researching how knowledge influences or is considered in policy and planning (Grove et al. 2024). The “shifts” capture and present the plurality of the ways that urban ecology is evolving and question the positioning of cities as ‘problems’ that need to be dealt with, areas of deprivation, and resource consumption distant from ecological value.

## Key learning points from a shift’s perspective in urban ecology

First, we live on an urbanized planet (Pickett et al. 2024). This means that urban impacts, attitudes, and demands—relationships—now reach well beyond the formal or perceived boundaries of cities and suburbs (Elmqvist et al. 2013). Across much of the world, villages, traditional agricultural and pastoral lands, and interfaces between wildlands and settlements are all changing (Berry 1990). Therefore, “urbanized” now means both the direct and distant or indirect effects of urbanization (Seto et al. 2012; Boone et al. 2014). Planetary urbanization has long been recognized in disciplines beyond ecology. Sociologist Henri Lefebvre Field (2003) asserted that in the 1970s, “The *urban fabric* grows, extends its borders, corrodes the residue of agrarian life. The expression, ‘urban fabric,’ does not narrowly define the built world of cities, but all manifestations of the dominance of the city over the country.” Contemporary scholars in many disciplines have documented conditions echoing Lefebvre in Europe (Schmid 2006; Gandy 2014), Asia (McGee 2014; Zhou et al. 2021), and Africa (Cilliers and Siebert 2012; McHale et al. 2013). Researchers attuned to long-distance connections involving urban places have shown that urban effects spread across regions and even among continents (Plowright et al. 2011; Seto et al. 2012; Liu et al. 2013). Urban ecology requires closer and more sustained contact with disciplines that range from culture to economy and employ diverse methods (Andersson et al. 2024).

Urban change is highly differentiated across the globe, across regions, and even within large cities (Nagendra et al. 2018; Frantzeskaki et al. 2024). For instance, there is both growth and shrinkage in urban places. Growth and spread may be particularly obvious in the Global South, but there are places in the already urbanized Global North that are spreading, while other districts in high-latitude urban places lose human and infrastructural density. Furthermore, many urban changes are accelerating. To engage in multiple forms of governance (much work is still embedded in a strong, top-down planning paradigm), urban ecology must help overcome several challenges arising from the social and biophysical heterogeneity of diverse urban systems and the knowledge that informs their development (Andersson et al. 2024). Indeed, early sociological explorations of the urban realm laid a foundation that emphasized the social heterogeneity of cities as opposed to the seeming homogeneity of rural life (Wirth 1938; Fitzi 2018). Social differentiation, specialization, alienation, and segregation are associated with such heterogeneity, and these phenomena often reflect the different power relationships in urban places. Not all power relationships are obvious, and tacit racism (Rawls and Duck 2020), caste (Center for Human Rights and Global Justice 2007), and class (Robertson and Westerman 2015) differences affect how different social groups experience environmental benefits, amenities, and hazards. It is a persistent challenge for planning and governance to achieve equity of opportunity and security from hazards, given the similarities of social hierarchies in urban situations around the world (Grove et al. 2024).

Second, cities were early forerunners of the Anthropocene—human activities are fundamentally involved in shaping urban nature, and without us, it would become something unknown, very different from what was there before the cities (e.g., Kowarik 2023; Hobbs et al. 2014; Andersson et al. 2022). With this degree of involvement, how we choose to do things is vital for the capacity and capability of urban nature to regenerate and for biodiversity to thrive. As long as we live in them, cities will never be ‘wild’, but nature and natural dynamics can be allowed more space. Holling and Meffe (1996), and many with them, have argued that there is a fallacy in ‘command and control’ in suppressing variation and enforcing (perceived) predictability. Making space for less human-controlled natural dynamics would mean a shift requiring careful thought and much research (e.g. VanMeerbeek et al. 2019; Frantzeskaki et al. 2024). Within current discourses on ecosystem services, green infrastructure, ecosystem-based adaptation, and nature-based solutions, there has been a tendency to overemphasize nature’s positive contributions to people (e.g., Lyytimäki et al. 2008; Ommer et al. 2022). While we rely on a functioning biosphere for our survival, it is its own thing, and overselling or pushing urban nature to be a service provider reinforces the philosophy of command and control. Our relations with nature might be harmful, both for humans and other species and making space for wilder nature in cities means we also need to rethink and manage differently how we interact with it. This might include differentiating access and use of different spaces (sharing instead of excluding), allowing and learning to appreciate variation and unpredictable change over time, and relations built on respect and reciprocity rather than utilitarianism (e.g., Salmón 2000). That is, we must attend to all sorts and outcomes of nature-people relationships while recognizing that different social groups and Global North and South places will experience different such relationships (Pickett et al. 2024; Grove et al. 2024). Partly, this is a challenge of reconciling notions of artificial and natural. In the Anthropocene cities, human-made things (and ideas) must not be seen as divorced from nature (Soga and Gaston 2016)—what we do has a bearing on nature, for good or bad, and to be close to us and our minds, nature needs to be present also in the ‘artificial’. Urban ecology has a role to play in engaging with biophilic design, investigating digital ecological literacy, developing extensive management practices, and so on. Cities are also where one of the most urgent concerns of the Anthropocene needs to be more effectively addressed: While cities drive change, they are also vulnerable to it. Climate change and other potential or already manifest hazards question both the morphology and layout of our cities. City form needs to be rethought and partly redone, and here, urban ecology has a role.

Third, working solutions, informed by urban ecology, must be systemic and transdisciplinary to be realistic and relevant (Kabisch et al. 2022; McPhearson et al. 2021b; Kowarik 2023). Solutions advancing urban sustainability, including nature-based solutions (Keeler et al. 2019), need to strive to reconnect people and nature (Sarabi et al. 2023), and recent research points out that this has been achieved in different contexts more effectively through transdisciplinarity. Urban nature-based solutions can be sourced and/or co-created on different scales and can vary depending on the socio-political context of cities that give ground to them. For their co-design and employment, multiple knowledges are required, making interdisciplinarity an imperative condition for building evidence, strengthening urban planning adaptation cycles, and ensuring that their social-ecological-economic performance is sustainable and provides just outcomes over time (Grove et al. 2024; Pickett et al. 2024). In this mix and web of knowledges, ecology plays an important role in ensuring that from the ideation and co-creation of solutions to their implementation, they incorporate ecological knowledge, safeguard how the ecology of places is to be protected, safeguarded, and restored concomitantly with the co-creation with place and space (Frantzeskaki et al. 2024).

The evidence for the impact and efficacy of urban 'solutions' needs interdisciplinary scrutiny to ensure that they do not deepen socio-ecological inequalities or generate ecological trade-offs. For this, we need to systematize the evidence (Esmail et al. 2022) and possibly examine it through new understandings, such as the shifts that pose new questions to progress research and practice (Andersson et al. 2024). Taking the urban ecology shifts as lenses to identify and/or source solutions, we may be able to understand evidence differently, build systemic understandings of actionable knowledge, and develop a science of synthesis, including clear, testable, and robust ways of weighing evidence and connecting perspectives. This also relates to the intentionality of urban ecology—with the shifts in urban ecology—to transform and become more actionable and relevant to policy, urban planning, and society (Frantzeskaki et al. 2024).

These twenty-first-century urban facts can inform action at its frontier with practice. Knowledge transfer and building site- and situation-specific knowledge must be able to handle different types of evidence and diverse relations between people and urban nature. Fundamental to this is a strong transdisciplinary practice. Specifically, transdisciplinarity in and of urban ecology needs to underpin the future branching out and bridging of pathways of urban ecology. Transdisciplinarity can account for people's ever-changing views, perceptions, and values for urban ecosystems and the interlinked SETS dynamics (McPhearson et al. 2021a). “Living” in urban situations is multifaceted, suggesting many arenas for action and partnership with practitioners and communities (Mitchell et al. 2015; Pickett et al. 2024). Living can mean to rely on, to understand, to shape, and to share. To actively involve science in these facets of urban life requires urban ecologists to (1) examine the direct and indirect impacts of urbanization, (2) quantify both local networks and teleconnections in which an urban place participates, and (3) reveal the reciprocal interactions of livelihood, lifestyle, and urban ecosystem conditions (Pickett et al. 2024). Consequently, action requires interdisciplinary intellectual foundations (Andersson et al. 2024) to support trans-disciplinary collaboration with communities (Grove et al. 2024). Transdisciplinarity can also be seen as a stepping stone for future urban ecology research, and urban ecology shifts can also prepare for an open inquiry and broader learning from stakeholders across sectors, moving away from instrumentalizing urban nature to co-create with urban nature.

Fourth, individuals' needs, aspirations, stories, and economies, as provided by the nature of the city's needs and how they shape SETS in cities, need to be examined. Future research of urban ecology and theoretical shifts in urban ecology need to allow for novel research architectures and designs to examine socio-ecological dynamics in the Anthropocene. Understanding equity and empathy, which emphasizes different social groups' exposure patterns to environmental benefits and burdens, is the first step toward environmental justice (Wolch et al. 2014). The concept of justice refers to several related mechanisms by which equity among heterogeneous social groups and rankings can be achieved. Justice can mean the fair spatial distribution of environmental hazards and benefits, the inclusion of all social ranks and groups in the process of planning and governance, the assurance that all participants are heard and therefore represented in the planning deliberations, and finally, justice can mean overcoming the persistent outcomes and legacies of past inequities and procedural exclusions (Grove et al. 2024). Any differential power relationships must be considered and resolved in the process (Campbell and Gabriel 2016; Heynen et al. 2018). Justice, therefore, needs to be a starting point conceptually and theoretically, for example, by integrating the principles of urban ecology and social-ecological justice. In this way, a transformative urban ecology paradigm will include ecological and social diversity and livability and will be in conversation with more perspectives, disciplines, and practices to strengthen a justice-centered discipline (Table 1).

**Table 1:** Key messages inspired from a shifts’ perspective in urban ecology

Key messages	New understandings	Implications (what is needed to act upon?)	New planning and governance challenges
#1 We live on an urbanized planet (ecology of cities)	Urbanization is not bounded to cities themselves and the ‘urban’ is now present nearly everywhere	Interdisciplinary, cross scale efforts are required for linking cities to complex global dynamics	Unpack and understand hidden power relations, urban planning to consider cross boundary governance
#2 Urban nature is hybrid, with yet to be explored opportunities and constraints (ecology of cities)	The hybrid nature of cities needs to be embraced more fully—nature needs (and has a right to) more space in cities	Variability and diversity need to be reintroduced in ways that do not cause unnecessary risks to people. In our expectations as well as in the cities themselves	Letting go of control, differentiating use, access and rights to the city, negotiating diverging needs, forming positive, reciprocal relations
#3 Transdisciplinarity is foundational for delivering inclusive social-ecological-technological solutions for living in harmony with nature in cities (ecology of cities)	Different knowledges and expertise are required to co-design solutions that strengthen people-nature relations	New ways of collecting evidence, formulating new critical questions to bring inter- and transdisciplinary teams together to avoid externalities, blind spots and injustices in the making	Improve position and intentionality of urban ecology to connect with policy and urban planning
#4 The ecology of the mind—attention to people’s relation to nature and the other-than-human (ecology with cities)	Urban nature is for people and other than humans, requiring new designs and stewardship approaches	New ways of thinking, relating and designing urban nature to recognize needs from multiple species (more-than-human)	Employ new approaches to account for the more-than-human needs in the urban environment

Taking the shifts as a lens, new themes for research are emerging. Our proposed future research agenda consists of two themes: First, reconceptualize urban resilience to take into account shifts in perspective; Second, working as a multi-disciplinary field toward a science of synthesis.

Urban nature must be resilient to global and local environmental change (McHale et al. 2015) and disturbances (Grimm et al. 2017) for it to be an ecological source of resilience (Pickett et al. 2024) for complex SETS experiencing multiple challenges (McPhearson et al. 2015; Andersson et al. 2022). If nature in urbanized regions is to deliver on the promises of nature-based solutions to provide social, economic, and infrastructure benefits reliably over time where most people live, then the ecological subsystems must themselves be able to persist and thrive in the face of multiple local, regional, and global pressures (Lin et al. 2021). Taking the four shifts as the starting point in urban ecology, a reconceptualization of urban resilience may be a future research pathway. Specifically, urban ecology needs to advance understanding of population, community, and ecosystem resilience in cities by asking questions such as: How can we ensure that the nature in nature-based solutions and its ecosystem functioning enhance resilience to rising heat, storms, drought, flooding and other extremes and human-induced disturbances in cities? How should we measure and evaluate urban ecological resilience in order to provide input for and with cities to meet urban sustainability agendas? As thus, a deeper understanding and assessment of the resilience of urban ecological infrastructure is critical to ensure that urban nature itself is resilient and managed to ensure it can deliver the nature-based solutions we need and that it underpins the ecological contribution to generalized urban SETS resilience (McPhearson et al. 2015). In relation to this, new research methods, including but not limited to citizen science, can be employed to evaluate the resilience of urban nature and to move beyond the narratives of loss [think of the seminal work of Half-Earth (e.g., Pimm et al. 2018)], while at the same time, balancing the eco-optimism of ecological civilization writings.

Finally, synthesis, and developing a science of synthesis, is particularly important given the current development of urban science and the extraordinarily dynamic nature of global urban changes (McHale et al. 2015; Gandy 2022). The changing urban world is beset by new patterns of urban transformation, new pressures on urban systems, new patterns and intensities of hazard, and new mechanisms of adjustment, adaptation and denial. The global diversity of urban form and change, and the immense number of fields beyond our own that are concerned with and contribute to urban thinking require the continued openness and varied mechanisms of synthesis.

We see synthesis as predominantly creative, and distinct from criticism or hypothesis testing. Our syntheses have sought commonality and complementarity across differences. Our syntheses started with organizing and understanding the nature of the cases, data, or ideas that we brought together. We have worked with various boundary objects (Star and Griesemer 1989) to accommodate contrasting viewpoints while allowing collaborators to explore conceptual identity across the viewpoints. Boundary objects are not the same as consensus models, as reaching consensus too early may silence difference (Bark et al. 2016) and exclude vulnerable voices (Langemeyer and Connolly 2020). Seeking commonality may involve meta-analysis (Furr et al. 2010) or discovering mechanisms of change rather than presumed ultimate or teleological causes (Robinson 2021). It may also emerge from new examples or cases that fill in previously missing or neglected causes (Partelow 2018).

More generally, our process has been supported by a culture of openness and exploration among the community seeking synthesis. Openness can exploit analogies and metaphors across disciplinary sutures, but there is a danger that the “common sense” of such imagistic thinking can become fixed and ultimately harm the assessment of fundamental assumptions and mechanistic thinking (Light 2009; Fairfield 2024). This last point emphasizes that a culture of creativity and openness can facilitate synthesis (Pickett 1999). The critical impulse can stifle needed creativity if it is employed too soon (Pickett et al. 1999). But as synthetic ideas and tools are exercised, a critical stance among the community of collaborators should become operative. Nevertheless, synthesizers and the culture and practices of synthesis must be nurtured in interdisciplinary and transdisciplinary work (Bammer 2013; Boone et al. 2020).
